# A single-institution experience with intraoperative *in vivo* confocal laser endomicroscopy for brain tumors in 50 patients

**DOI:** 10.3389/fonc.2025.1565935

**Published:** 2025-05-14

**Authors:** Yuan Xu, Thomas J. On, Irakliy Abramov, Oscar Alcantar-Garibay, Joelle N. Hartke, Jennifer M. Eschbacher, Kivanc Yangi, Michael T. Lawton, Kris A. Smith, Randall W. Porter, Mark C. Preul

**Affiliations:** ^1^ The Loyal and Edith Davis Neurosurgical Research Laboratory, Department of Neurosurgery, Barrow Neurological Institute, St. Joseph’s Hospital and Medical Center, Phoenix, AZ, United States; ^2^ Department of Neurosurgery, Barrow Neurological Institute, St. Joseph’s Hospital and Medical Center, Phoenix, AZ, United States; ^3^ Department of Neuropathology, Barrow Neurological Institute, St. Joseph’s Hospital and Medical Center, Phoenix, AZ, United States

**Keywords:** brain tumor, confocal laser endomicroscopy, fluorescence-guided surgery, glioma, intraoperative diagnosis, neuro-oncology, neurosurgery

## Abstract

**Objective:**

Confocal laser endomicroscopy (CLE), a handheld imaging technology, provides intraoperative real-time cellular resolution examination of tissue architecture. We evaluated the feasibility and diagnostic capability of the first clinically approved CLE system for intraoperative *in vivo* imaging of brain tumors.

**Methods:**

A total of 50 patients who were to undergo brain tumor surgery were prospectively enrolled. CLE images were interpreted by one CLE-experienced neuropathologist as lesional, non-lesional, or non-interpretable and compared to tissue histology acquired at the same location under neuronavigation. Diagnostic accuracy of CLE imaging was calculated using permanent sections as the standard for comparison. The neuropathologist provided real-time image interpretation using a built-in telepathology consultation platform in 27 cases.

**Results:**

The final pathology of the tumors in these patients included 28 gliomas, 5 meningiomas, 3 metastatic brain tumors, 5 treatment-related changes, and 10 other primary intracranial tumors. A total of 13,535 interpretable images were acquired from 304 regions of interest (ROIs). The first informative images were acquired within 10.5 s after the initiation of CLE imaging for each ROI. Mean CLE imaging time per case was 8.6 min. Using telepathology consultation extended CLE imaging per case time by 3.8 min (p=0.005). Communication between neurosurgeon and neuropathologist lasted 3.9 min per ROI. Overall sensitivity and specificity of CLE imaging were 93% and 81%, respectively. The specificity differed significantly between core and margin ROIs in glioma cases (93% vs. 50%, p=0.039). Diagnostic performance was not statistically different between new and recurrent glioma cases or between glioma and other tumor types.

**Conclusions:**

The clinically approved CLE system allows intraoperative *in vivo* visualization of tissue histoarchitecture and identification of lesional tissue in real time, without the need for tissue biopsy and processing. CLE is efficient and cost-effective, with high diagnostic accuracy at the glioma core. However, CLE imaging at the tumor margin remains challenging.

## 1 Introduction

Confocal laser endomicroscopy (CLE) uses a handheld probe to deliver real-time intraoperative imaging of tissue histoarchitecture during intracranial neurosurgical procedures (1). The current CLE system uses fluorescein sodium (FNa) as a contrast agent, enabling digital biopsies without the need for tissue resection or processing (2). This technology presents significant potential as a tool for intraoperative guidance and brain tumor interrogation, particularly for assessing tumor margins for cellular infiltration (3).

Following animal model (4, 5) and *ex vivo* human brain tumor studies (6, 7), the clinical-grade CLE system (CONVIVO *In Vivo* Pathology Suite, Carl Zeiss Meditec AG, Jena, Germany) was cleared by the US Food and Drug Administration for clinical neurosurgery applications (8). We subsequently conducted the first clinical study of CLE, involving 4 neurosurgical teams and 30 patients, to validate the safety, feasibility, and accuracy of CLE correlated with permanent histological sections (9). A telepathology software platform (TSP) was introduced during the study, enabling real-time intraoperative neuropathology consultations (10, 11).

In this study, we report the findings from an additional 20 patients enrolled in the complete CLE clinical study, increasing the total number of patients who have undergone CLE-assisted brain tumor resection at our institution to 50. This second phase of the study focused on intrinsic brain tumors, particularly gliomas, to assess the accuracy of tumor margin identification with CLE and the combined use of FNa with 5-aminolevulinic acid (5-ALA). The TSP consultation platform was used in 27 of the 50 cases. We also analyzed the time efficiency and cost-effectiveness of CLE, 5-ALA, and frozen section diagnostic methods.

## 2 Methods

### 2.1 Study design

A prospective feasibility study of the clinical CLE system was conducted at Barrow Neurological Institute at St. Joseph’s Hospital and Medical Center in Phoenix, Arizona, following approval from the Institutional Review Board for Human Research (IRB No. 19-500-403-80-12). The study focused on evaluating the CLE system during *in vivo* brain surgery. Patients were enrolled from May 2020 to October 2023. In the study’s first phase, 30 patients scheduled for brain tumor surgery were consecutively enrolled, regardless of preoperative diagnosis, for a clinical feasibility study (9). The criteria became more specific in the study’s second phase, focusing on individuals with intrinsic brain tumors and limiting the study population to 20 patients. Exclusion criteria included renal failure, pregnancy, breastfeeding, age <18 years, previous adverse reactions or hypersensitivity to FNa, and inability to provide informed consent. The surgeons adhered to their standard practices for tumor removal. CLE was not used to guide intraoperative clinical decision-making. The data supporting this study’s findings are available from the corresponding author upon reasonable request and institutional review board approval, as applicable.

The study aimed to 1) assess the safety of the CLE system in the operating room; 2) evaluate the system’s usability and the surgeon’s ability to obtain interpretable CLE images during surgery; and 3) correlate *in vivo* CLE images with traditional histopathology and neuronavigation data. Primary endpoints included 1) ensuring that the number of adverse effects (e.g., tissue injury or postoperative infections) during cases involving the CLE system did not exceed the historical control for similar patients in whom CLE was not used and 2) confirming that the neurosurgeon could operate the CLE system to obtain noninvasive digital (i.e., optical) biopsies in 90% of cases. Secondary endpoints consisted of 1) having CLE imaging data interpreted as “normal” or “abnormal” tissue by a neuropathologist in over 80% of cases; 2) achieving agreement between multiple biopsy assessments of CLE data interpretation and histopathological evaluations in 80% of biopsies, along with recorded neuronavigation positions indicating biopsy locations; and 3) on average, allowing the neurosurgeon to obtain a diagnostic image within 10 min of beginning *in vivo* CLE imaging (i.e., about half the time typical for the fastest frozen section process).

### 2.2 CLE imaging and tissue biopsy acquisition

Five neurosurgical teams carried out the surgical procedures. Each team included an experienced attending neurosurgeon familiar with CLE, who served as the primary user. Residents lacking experience with the CLE probe operated the device under supervision. Neurosurgeons viewed the CLE images on the monitor in real time within the operating room.

A dose of 5 mg/kg FNa was administered 5 min before CLE imaging of the first region of interest (ROI) in the case. Each surgeon determined the number and locations of ROIs at their discretion, considering procedural safety and comfort level. Frozen section biopsy specimens were obtained as needed by the operating surgeon. Immediately after CLE imaging, the operating neurosurgeon collected tissue biopsy specimens at the imaged ROIs if it was deemed safe, based on their sole discretion. All frozen and permanent biopsy specimens were processed and stained with hematoxylin and eosin. The surgical and imaging protocol allowed for up to nine different ROIs to be imaged within the tumors and masses. The ROIs were categorized as either tumor core or tumor margin based on their location as seen on intraoperative magnetic resonance imaging (MRI) neuronavigation. For both high- and low-grade gliomas and other tumors or masses, optical and tissue biopsies were taken from up to three locations within the tumor core. Areas at the tumor margin of gliomas were targeted for up to four optical and tissue biopsies, while up to three areas of distant infiltrative tumor margins were also selected for optical and tissue biopsies.

Optimal CLE image acquisition parameters have been described previously (12). TSP was used for real-time intraoperative neuropathology consultation in cases in which the system was available. A total of 16 patients underwent CLE FNa imaging simultaneously with 5-ALA wide-field operating microscope fluorescence imaging. To prevent any potential bias that 5-ALA fluorescence may introduce in the interpretation of CLE images, the CLE imaging ROI was assessed immediately after CLE imaging using 5-ALA-induced fluorescence and the surgical microscope’s blue light fluorescence filter. A comparison of CLE and 5-ALA fluorescence imaging was conducted and is presented in a separate study (13).

### 2.3 CLE image interpretation and diagnostic performance

CLE images from cases involving intraoperative TSP consultation were interpreted in real time by a neuropathologist experienced with CLE imaging. The same neuropathologist later interpreted CLE images from cases without TSP consultation after the case was completed. For each case, the neuropathologist was provided with information on the lesion location, MRI appearance, and previous treatment (for recurrent cases). CLE images from each ROI were classified as lesional, non-lesional, or non-interpretable. ROIs with non-interpretable CLE images were excluded from the analysis. The diagnostic performance of CLE imaging, including sensitivity, specificity, positive predictive value (PPV), and negative predictive value (NPV), was calculated using permanent histology from location-matched specimens as the gold standard. All ROIs from glioma cases were further stratified based on location (core vs. margin) and treatment status (newly diagnosed vs. recurrent).

### 2.4 Time and cost-effectiveness

Efficiency and cost-effectiveness were compared between CLE and the two most common intraoperative diagnostic methods: frozen section biopsy and 5-ALA wide-field fluorescence imaging using the operating microscope. Efficiency metrics included time allocated for TSP per ROI and per case and the duration from tissue acquisition to the availability of a frozen report. For the cost analysis, we included all expenses related to intraoperative diagnostics. The cost of CLE imaging was calculated on the basis of the assumption that it would be used for five cases per week over a 5-year period.

### 2.5 Statistical analysis

Statistical analyses were performed using GraphPad Prism 9 (GraphPad Software, Inc.). Continuous variables are presented as mean (SD), while categorical variables are presented as counts and percentages. Times spent on intraoperative diagnostics were compared using Welch’s *t*-test. Diagnostic accuracies were assessed using a two-proportion Z-test. Sensitivities, specificities, PPVs, and NPVs were calculated from standard formulas using 2×2 contingency tables and statistically compared using the chi-squared test; p-values of <0.05 were considered statistically significant.

## 3 Results

### 3.1 Descriptive analysis

From May 2020 to October 2023, 50 patients were enrolled (male/female, 24:26; p=0.78). The first 30 patients were enrolled consecutively to evaluate the feasibility and safety of the CLE system, and the results were published in a previous paper (9). All but 2 of the last 20 patients had a preoperative diagnosis of glioma and were included for a focused analysis of CLE imaging in the most common type of intra-axial brain tumor. The mean age at presentation was 53.5 (range, 23–84) years. A total of 12 patients exhibited non-enhancing mass lesions on preoperative MRI. One patient had two discrete contrast-enhancing masses. The 51 mass lesions included 28 gliomas, 5 meningiomas, 10 other central nervous system tumors, 3 metastatic brain tumors, and 5 treatment-related changes that were negative for tumors (**Table 1**). No adverse effects attributable to intravenous FNa administration, oral 5-ALA administration, or intraoperative CLE imaging were identified. In addition, no surgical site infections related to CLE probe use were reported.

**Table 1:** Final pathological diagnoses of 51 brain tumors in 50 patients.

Primary CNS tumor	Tumor type (n)
Glioma (n=28)	
WHO grade 1	Pilocytic astrocytoma (2), subependymoma (1)
WHO grade 2	Oligodendroglioma (1)
WHO grade 3	Astrocytoma (4), oligodendroglioma (2), recurrent oligodendroglioma (1), HGAP (1)
WHO grade 4	Glioblastoma (7), recurrent glioblastoma (7), astrocytoma (1), recurrent astrocytoma (1)
Meningioma (n=5)	
WHO grade 1	Meningioma (3)
WHO grade 2	Atypical meningioma (2)
Other CNS tumor types (n=18)	
CNS metastatic tumors	Breast carcinoma (1), lung adenocarcinoma (1), renal cell carcinoma (1)
Treatment-related changes negative for tumors	Gliosis, necrosis, and other changes (5)
Other miscellaneous CNS tumors	Glioneural and neuronal tumor (2), hemangioblastoma (2), vestibular schwannoma (2), pineocytoma (1), choroid plexus papilloma (1), perineurioma (1), mature teratoma (1)

CNS, central nervous system; HGAP, high-grade astrocytoma with piloid features; WHO, World Health Organization.

### 3.2 Intraoperative CLE imaging

**Figure 1:**
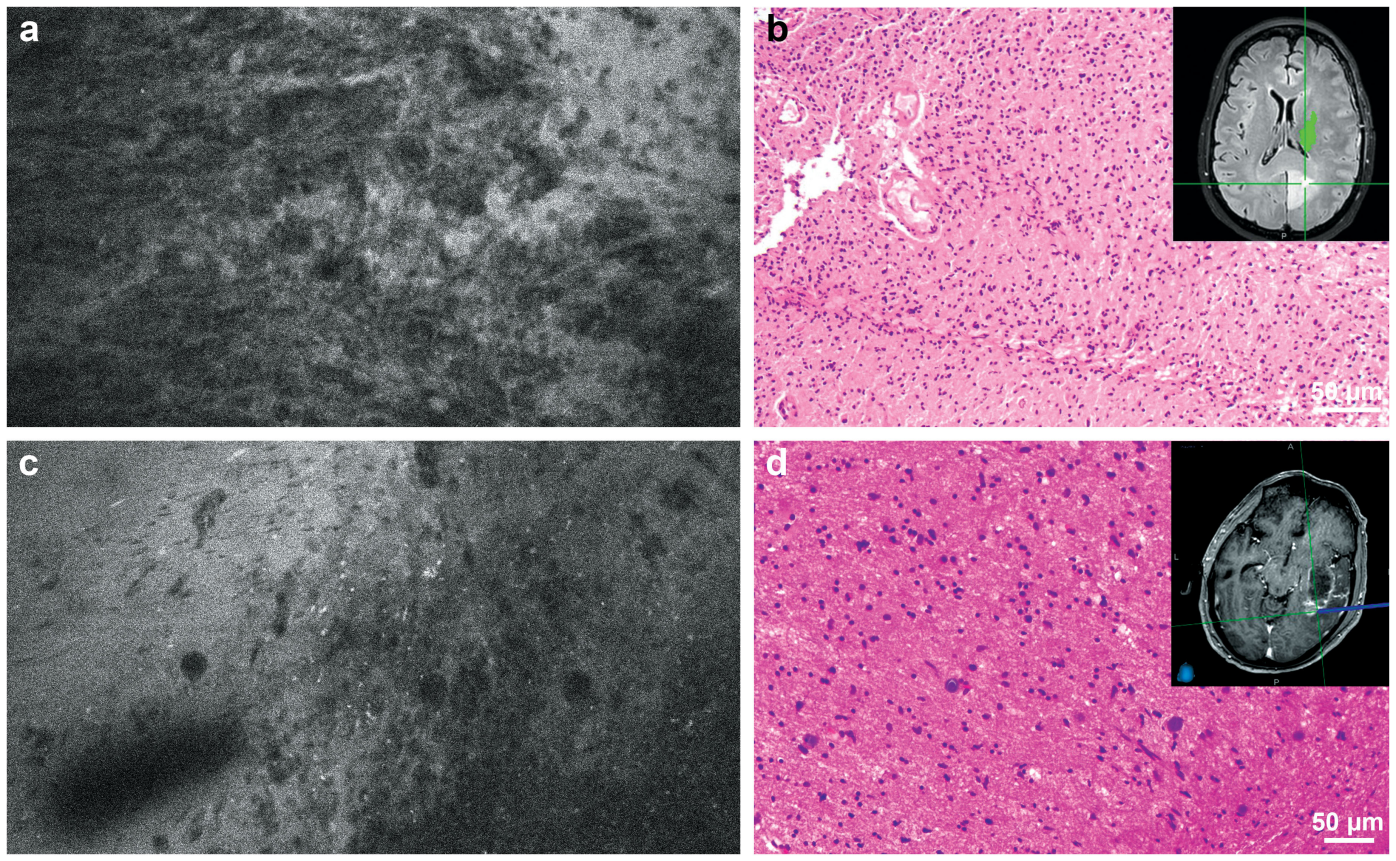
**(a)** Confocal laser endomicroscopy (CLE) image and **(b)** matching hematoxylin and eosin (H&E)-stained permanent histological section from the tumor core. Inset shows intraoperative neuronavigation. The CLE image was acquired at the core of a medial occipital mass that did not enhance significantly on preoperative magnetic resonance imaging and showed significant hypercellularity and pleomorphism. The histological section revealed a high-grade glial tumor without significant necrosis or microvascular proliferation. Isocitrate dehydrogenase (IDH) mutation analysis confirmed that this tumor was a glioblastoma, IDH-wild type, WHO grade 4. **(c)** CLE image and **(d)** matching H&E-stained permanent histological section from the tumor margin. Inset shows intraoperative neuronavigation. The CLE image acquired at the posterolateral margin of an enhancing lesion showed hypercellularity, indicating tumor infiltration, which was confirmed with histological section. Used with permission from Barrow Neurological Institute, Phoenix, Arizona.

Interpretable CLE images were acquired from all 50 patients. In total, 157 ROIs were examined [mean (SD) 3.1 (1.4) ROIs per case]. For all ROIs, the mean (SD) time interval between FNa administration and CLE imaging was 40.1 (42.3) min. The median (IQR) CLE imaging time per case was 8.3 (range, 5.5-12.7) min. Of the 157 ROIs examined, 142 produced 13,493 interpretable images [mean (SD) 95.0 (77.8) interpretable images per ROI]. On average, the first interpretable images were acquired within 10.5 (25.3) s after the start of CLE imaging for each ROI. The five neurosurgical teams completed 27, 13, 5, 3, and 2 cases, with total image acquisition times of 276, 109, 30, 18, and 15 min, respectively. A total of 114 ROIs were examined in 33 cases with a preoperative diagnosis of glioma. Among these, 59 were in the tumor core, and 55 were in the tumor margin (**Figure 1**).

**Figure 2:**
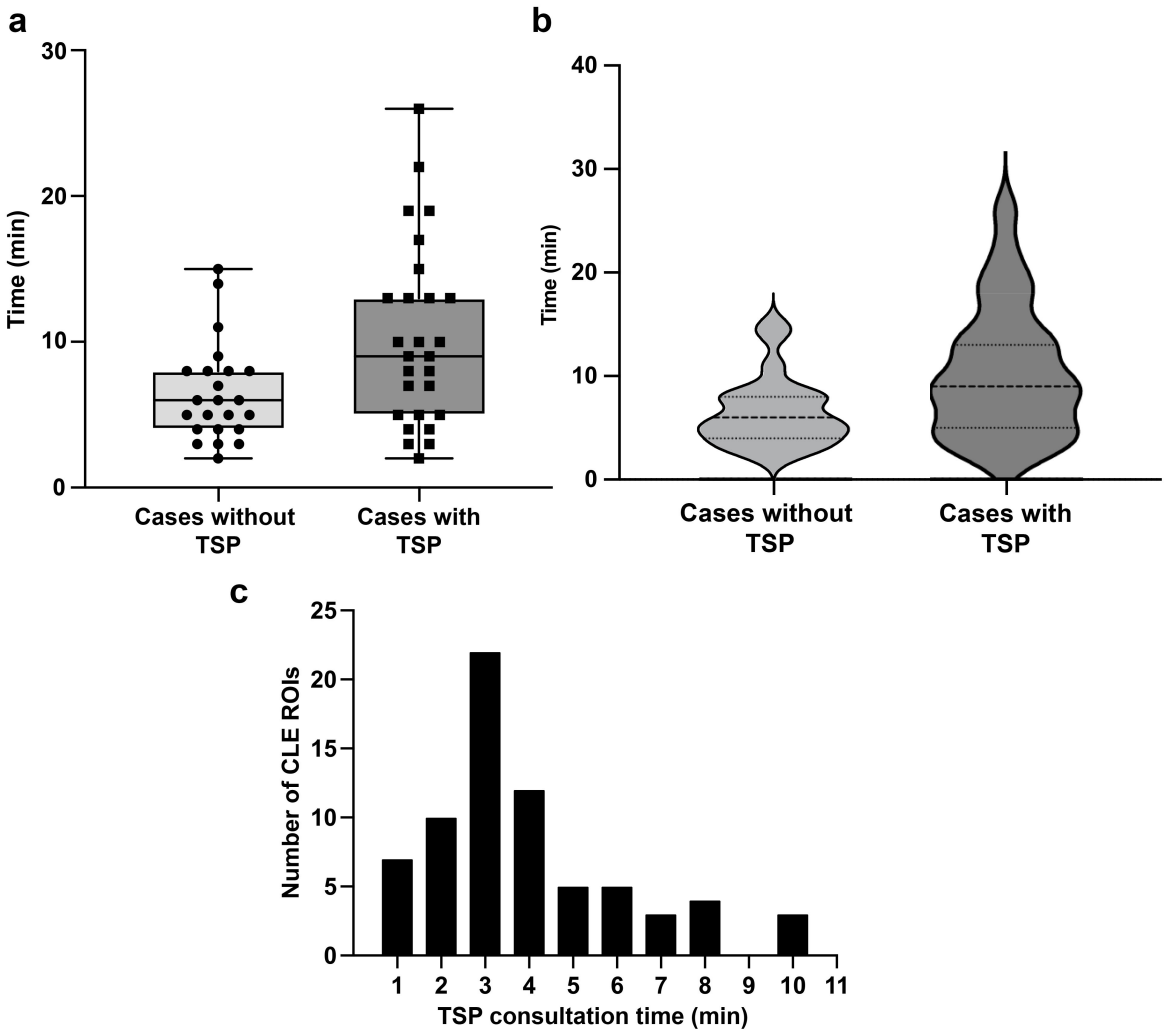
Time spent on confocal laser endomicroscopy (CLE) imaging. **(a)** Box and whisker plot showing CLE imaging time per case with and without telepathology software platform (TSP) consultation. The horizontal line in the middle of the box indicates the median, the top and bottom borders of the box indicate the 75th and 25th percentile, respectively, and the top and bottom whisker indicate the range; the black circles and squares indicate individual values. **(b)** Violin plot showing CLE imaging time per case, indicating that TSP consultation significantly increased CLE imaging time. The dashed line indicates the median CLE imaging time per case, and the upper and lower dotted lines indicate the 25th and 75th percentile. **(c)** Distribution of time spent on TSP consultation for each region of interest (ROI), with a mean (SD) of 3.9 (2.2) min. Used with permission from Barrow Neurological Institute, Phoenix, Arizona.

Consultations using the TSP were conducted during CLE imaging of 71 ROIs in 27 of 50 patients. On average, the time spent on CLE imaging per case without TSP consultation was 6.5 (3.4) min. In contrast, the mean (SD) CLE imaging time per case with TSP consultation was 10.3 (6.2) min (**Figure 2A**). Using TSP consultation increased the average CLE imaging time per case by 3.8 min (p=0.008; **Figure 2B**). Communication between the neurosurgeon and the neuropathologist lasted 3.9 (2.2) min per ROI (**Figure 2C**). An audio connection is not integrated into the system. Therefore, voice communication must occur through a separate line of communication or by the annotation of the image by the neurosurgeon or neuropathology station.

### 3.3 CLE diagnostic performance

A total of 103 tissue biopsy specimens were obtained from 50 patients (mean, two specimens per patient; range, 1–6). The neuropathologist matched the CLE images with histology in all but one case (98%), which was a perineurioma. The diagnostic performance of CLE imaging is shown in **Table 2**. The overall sensitivity and specificity of CLE imaging were 93% and 81%, respectively. Subgroup analysis showed consistently high sensitivity (>85%) and PPV (>80%) across all subgroups and revealed a statistically significant difference in specificity between core and margin ROI groups (93% vs. 50%; p=0.04). In addition, tumor infiltration was successfully ruled out in 8 out of 10 ROIs imaged with CLE in cases of treatment-related changes that are negative for tumor. Compared to permanent histological biopsy results, the diagnostic accuracy rates of CLE imaging and frozen section biopsy results did not statistically differ across all specimens and glioma specimens (**Table 3**).

**Table 2:** Grouped analysis of intraoperative confocal laser endomicroscopy imaging diagnostic accuracy compared with tissue stained with hematoxylin and eosin as the gold standard.

Parameter	Glioma	Glioma core	Glioma margin	Recurrent glioma	New glioma	Non-glioma
Sensitivity	91 (83–98)	94 (81–99)	85 (64–95)	89 (67–98)	92 (78–97)	95 (75–100)
Specificity	77 (60–95)	93 (69–100)	50 (22–78)	78 (55–91)	75 (30–99)	100 (44–100)
PPV	91 (83–98)	97 (85–100)	81 (60–92)	80 (58–92)	97 (85–100)	100 (82–100)
NPV	77 (60–95)	87 (62–98)	57 (25–84)	88 (64–98)	50 (19–81)	75 (30–99)
Diagnostic accuracy (%)	87	94	75	83	90	95

Data are presented as percentage (95% CI), unless otherwise noted.

PPV, positive predictive value; NPV, negative predictive value.

**Table 3:** Comparison of diagnostic accuracy between intraoperative CLE imaging and frozen section with conventional histological hematoxylin and eosin staining as the gold standard.

Parameter	All specimens		Gliomas	
	CLE	Frozen section	CLE	Frozen section
Sensitivity	92 (83–96)	94 (79–99)	91 (83–98)	96 (80–100)
Specificity	80 (61–91)	75 (41–96)	77 (60–95)	75 (41–96)
PPV	93 (85–97)	94 (79–99)	91 (83–98)	92 (76–99)
NPV	77 (56–89)	95 (41–96)	77 (60–95)	86 (49–99)
Diagnostic accuracy (%)	89	90	87	91

Data are presented as percentage (95% CI), unless otherwise noted.

CLE, confocal laser endomicroscopy; NPV, negative predictive value; PPV, positive predictive value.

### 3.4 Efficiency and cost-effectiveness of intraoperative CLE imaging

**Table 4** shows the gross cost and cost per case of intraoperative CLE imaging. The cost per case of CLE imaging encompasses the purchase cost of the CLE system, the monthly subscription for the TSP consultation platform, one sterile sheath per case, one vial of 500 mg FNa for injection per case, and operating room time calculated based on the mean CLE imaging time per case in our study. The costs were calculated on a 5-year, five-cases-per-week basis. The estimated cost of three frozen section biopsies per case and wide-field fluorescence imaging with 5-ALA were included for comparison.

**Table 4:** Cost estimate (US dollars) of three intraoperative diagnostic methods.

Item	Gross cost	Cost per case
Intraoperative CLE imaging*		1,276
CLE system purchase	290,000	223
TSP consultation platform monthly subscription	940 per month	43
Sterile probe sheath	185	185
FNa injection (500-mg vial)	75	75
OR time	5,000 per hour	750
Frozen section biopsy†		1,050
Tissue processing per specimen	200	600
Neuropathologist time per specimen	150	450
Wide-field fluorescence imaging with 5-ALA (1,500-mg vial)	3,350	3,350

*The cost of intraoperative CLE imaging is calculated on a 5-year, five-cases-per-week basis.

†The cost of three specimens per case is shown for frozen section biopsy.

5-ALA, 5-aminolevulinic acid; CLE, confocal laser endomicroscopy; FNa, fluorescein sodium; OR, operating room; TSP, telepathology software platform.

## 4 Discussion

### 4.1 CLE image acquisition

**Figure 3:**
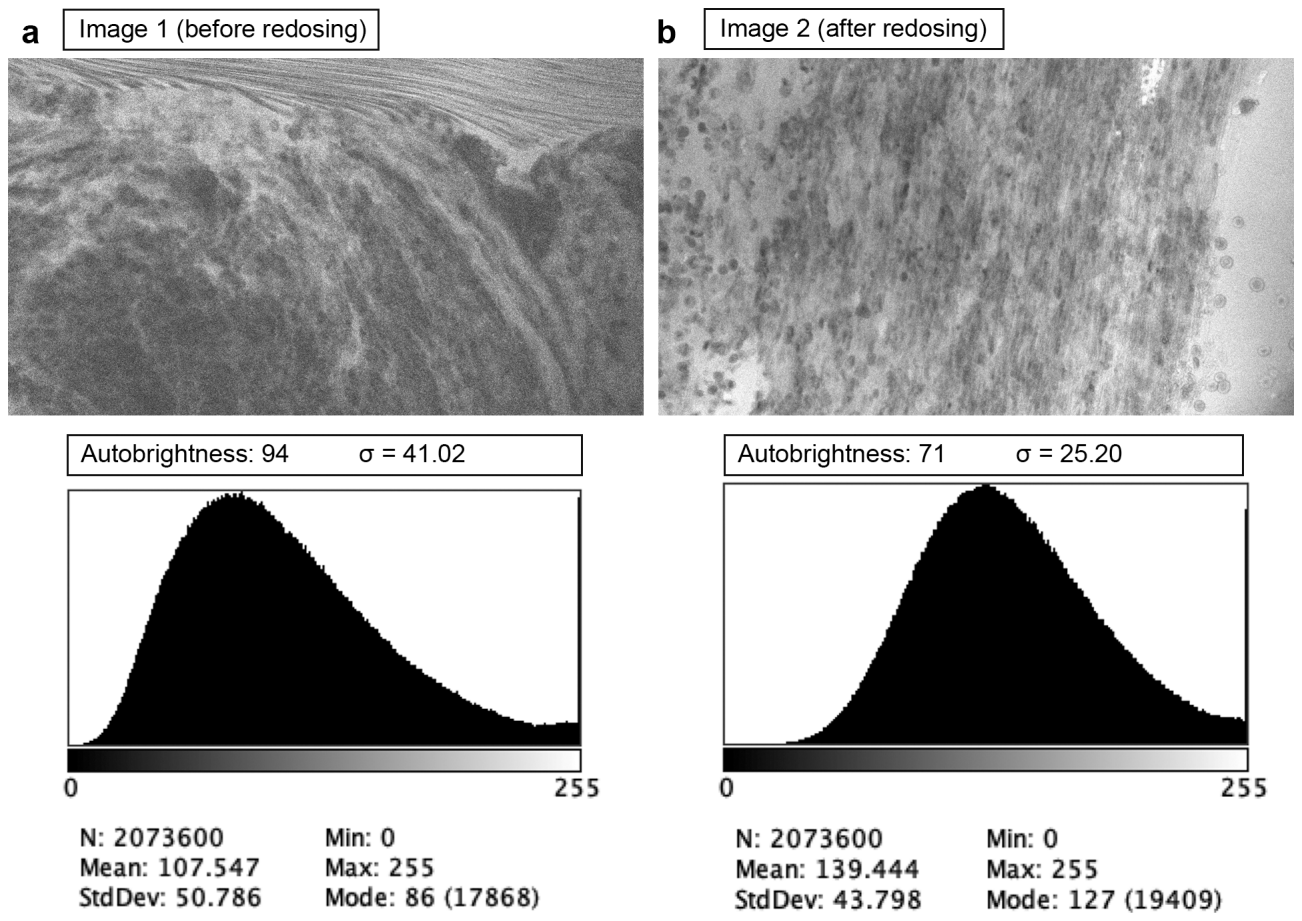
Examples of confocal laser endomicroscopy images acquired before **(a)** and after **(b)** fluorescein sodium redosing. Before redosing, high autobrightness compensated for the low fluorescence, making the image noisy (higher wavelet noise standard deviation σ). Used with permission from Barrow Neurological Institute, Phoenix, Arizona.

CLE offers the capability to assess tissue histology *in vivo* before tissue removal. Under optimal conditions, this process can be performed within seconds after FNa administration and imaging initiation. The pharmacokinetics of FNa make it ideal for CLE imaging. A 5-mg/kg intravenous bolus of FNa is administered minutes before imaging. Based on our observations, within seconds after injection, FNa fluorescence signal can be detected in tumor tissue with CLE. Although FNa is rapidly metabolized by glucuronidation in the liver, the metabolite is also weakly fluorescent. It has a half-life of more than 4 h, contributing to the extended CLE imaging window (14). Our previous analysis showed that image brightness and contrast do not significantly decrease during the first 2 h of imaging with proper laser power adjustment and with the use of the auto-brightness function (15). Restelli et al. published their prospective protocol for 75 intracranial tumors of various pathologies (16), and they recently reported the results of their study (17). In their protocol, FNa was administered at the induction of anesthesia, and wide-field fluorescence was used for tumor resection guidance. With wide-field imaging, this protocol leverages the enhanced permeability and retention effect to maximize fluorescence contrast between normal brain and tumor tissue (18). Whether this protocol improves tumor-to-normal-brain differentiation has yet to be demonstrated. In our protocol, positive fluorescence is not observed in normal brain tissue using CLE imaging, even shortly after FNa dosing, due to the intact blood–brain barrier in normal brain tissue. However, an extended delay could reduce CLE image quality, necessitating a second dose of FNa in certain cases. In one of our cases, a second 5 mg/kg dose of FNa was deemed necessary by the operating surgeon and administered 111 min after the initial dose. Before redosing, the images exhibited noticeable noise because of a weakened fluorescence signal and auto-brightness compensation. The second dose enhanced fluorescence and improved image quality, as shown by direct wavelet transformation noise estimation (**Figure 3**).

All goals and both primary and secondary endpoints were met and exceeded in our study. On average, the five neurosurgeons obtained interpretable and actionable CLE images within 10.5 s of scanning tissue. Similar to other reports, our study found that more than half of the acquired images were not interpretable, most commonly due to image distortion caused by motion artifacts or obscuration by clumped red blood cells (2, 6). We selected the scanning mode to generate the highest possible resolution, capturing an image every 1.3 s. This slow scanning speed contributed to the motion artifacts encountered during imaging. Movement in the tissue relative to the probe or disruption of the fiber-optic cable connecting the probe to the console led to motion artifacts that distorted part or all of the image. The imaging speed also limited the use of CLE as an intraoperative guidance tool to inspect a large area quickly. Generating interpretable images while moving the probe across a tissue surface requires a significantly higher scanning speed (19).

### 4.2 Intraoperative examination of tumor histology using TSP consultation

The built-in TSP connects the neurosurgeon and neuropathologist through a secure internet connection, allowing the neurosurgeon to share relevant clinical and surgical information while the neuropathologist reviews and interprets CLE images in real-time as they are acquired (10). The neuropathologist can connect from any location with a stable internet connection, although a slight delay and reduced image quality may occur. This level of access and availability is especially beneficial for hospitals lacking a neuropathology service or for cases in which evaluation of frozen sections must be completed off-site (20). Additionally, a continuous stream of CLE images provides more information than separate still images (10). This is crucial when differentiating tissue from background fluorescence or red blood cells from dense tumor cells.

TSP was used in 27 of the 50 patients. In procedures in which TSP was used, the mean CLE imaging time increased by 58% (6.5 min for cases without TSP consultation vs. 10.3 min with TSP consultation). Although the study protocol stated that no decisions were made on the basis of CLE imaging results, the neuropathologist provided valuable and surgically actionable information during the communication. For example, during a case involving an avidly enhancing sellar region tumor compressing the optic chiasm, the neuropathologist confirmed the diagnosis of hemangioblastoma through CLE imaging. This type of tumor poses a risk for significant or even catastrophic bleeding from biopsy or debulking procedures. Armed with this information, the operating neurosurgeon successfully decompressed both optic nerves and the optic chiasm by circumferentially dissecting the tumor from the surrounding neurovascular structures. This situation underscores the potential benefits of noninvasive histology assessment technology on the overall workflow. The absence of a built-in audio communication system between the neurosurgeon and neuropathologist should be addressed for optimal voice communication (10, 11).

### 4.3 Diagnostic accuracy of CLE imaging

In our study, the overall diagnostic accuracy of CLE imaging (89%) was comparable to that reported elsewhere (2, 9) and similar to that of frozen section biopsy (90%, p=0.87; **Table 3**). Although the low sample size of frozen section biopsies limited grouped analysis, the neuropathologist accurately diagnosed all 15 tissue specimens from glioma margins. In their recently published study, Wagner et al. reported higher overall diagnostic accuracy (95%) (21), although they did not specify the imaging location within the tumors (i.e., core or margin). In our *post-hoc* analysis of CLE imaging, we found that CLE at glioma tumor core ROIs yielded high accuracy (94%). In contrast, the diagnostic accuracy was lower at glioma margin ROIs (75%, p=0.03). This result aligns with our previous findings that focused on CLE imaging at glioma margins (3). Specifically, CLE demonstrates high sensitivity and PPV at glioma margins but low specificity and NPV (22).

The margin of infiltrative gliomas represents a mixed composition of tumor cells, inflammatory cells, and both affected and unaffected brain tissue. These areas become increasingly challenging to interpret due to the nonspecific nature of FNa staining (i.e., background staining), which accumulates in the extracellular space where the blood–brain barrier is compromised. We suggest that the lack of cellular uptake and specific interactions between FNa and cellular components impedes the effective differentiation of tumor from non-tumor cells. This differentiation is vital for optimizing the surgical resection margin of primary invasive tumors, where decisions about the extent of resection will be made. CLE imaging was intended to make a significant contribution to surgical digital optical histology at tumor margins, especially for gliomas. Further development of specific fluorophores compatible with the CLE laser is essential.

CLE revealed certain tumor-specific features in selected cases. These features were best seen in tumors with substantial FNa extravasation, such as meningiomas, metastatic tumors, and contrast-enhancing high-grade gliomas. Intrinsic brain tumors (e.g., gliomas, glioneuronal tumors, and neuronal tumors) appear the most heterogeneous with CLE imaging, depending on tumor grade, contrast enhancement, and imaging location.

**Figure 4:**
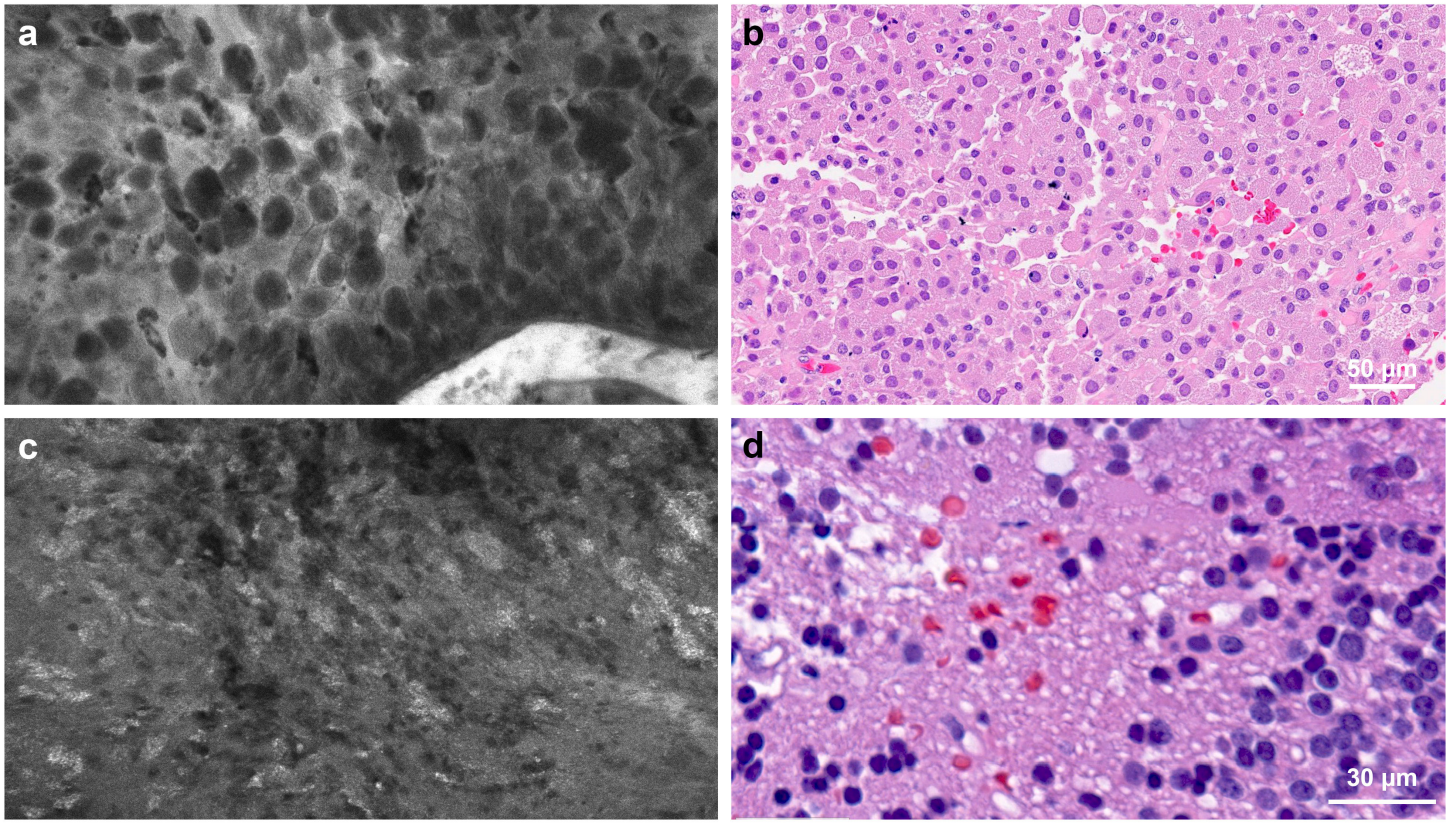
Confocal laser endomicroscopy (CLE) captured tumor histological features that correspond to hematoxylin and eosin (H&E)-stained permanent histology. **(a)** CLE imaging and **(b)** H&E-stained specimen showing large granular tumor cells in a patient with sellar granular cell glioblastoma. **(c)** CLE imaging and **(d)** H&E-stained specimen from a patient with recurrent glioblastoma showing a fluorescence signal from cellular uptake of fluorescein sodium, most likely by immune cells. This image type is less commonly seen in untreated patients. Used with permission from Barrow Neurological Institute, Phoenix, Arizona.

**Figure 5:**
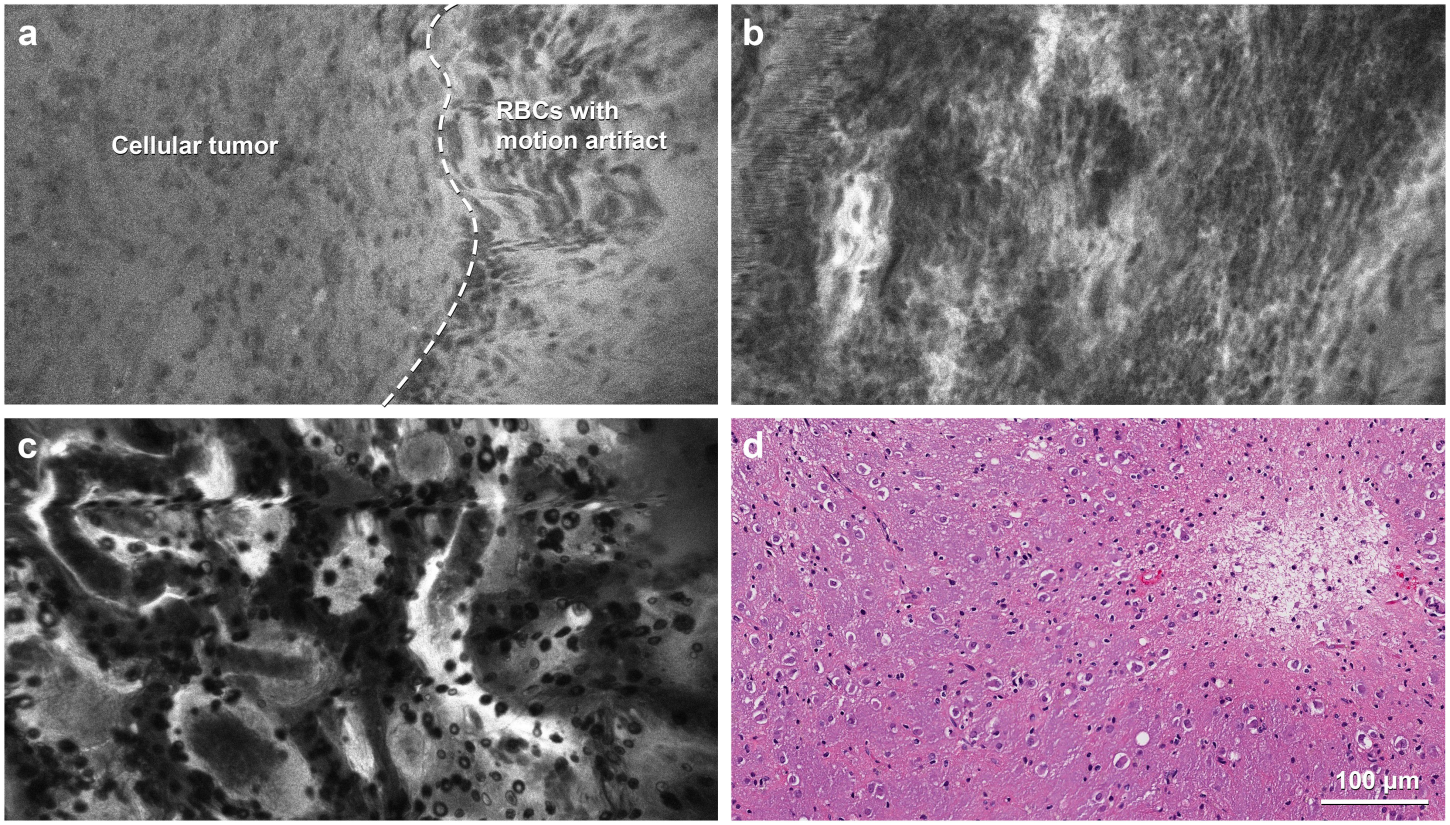
Confocal laser endomicroscopy (CLE) images in a rare case of multinodular and vacuolating neuronal tumor in the thalamus. **(a)** The portion of the image to the left of the dashed line, with a slightly darker background, is consistent with a low-grade tumor with mildly elevated cellularity, whereas the brighter half, to the right of the dashed line, shows motion artifacts that resulted from incomplete contact of the probe with the tissue. **(b)** CLE imaging at another region of interest shows more hypercellularity, suspicious for a more aggressive tumor. **(c)** CLE imaging shows the features of a normal choroid plexus in the third ventricle. **(d)** Hematoxylin and eosin staining shows tumor cells with neuronal features embedded in a vacuolated background without mitotic activity. RBC, red blood cell. Used with permission from Barrow Neurological Institute, Phoenix, Arizona.

CLE exhibited dense tumor cells, cellular atypia, and various histological features in contrast-enhancing high-grade tumors. For example, giant granular cells were identified in the CLE images from a unique case of the granular cell variant of glioblastoma (**Figures 4A, B**). Images depicting tissue treatment effects in recurrent gliomas are easily differentiated from tumor tissue. Fluorescence signaling from cellular uptake of FNa, likely by immune cells, was typically observed in previously treated patients but was less frequently observed in untreated patients (**Figures 4C, D**) (23, 24). However, in a non-enhancing multinodular and vacuolating neuronal tumor in the thalamus, CLE imaging at two separate ROIs revealed distinct histological patterns. Images from the first ROI showed characteristics of a mildly hypercellular tumor, while images from the second ROI showed markedly increased cellularity, raising suspicion for a higher tumor grade (**Figure 5**) (25).

**Figure 6:**
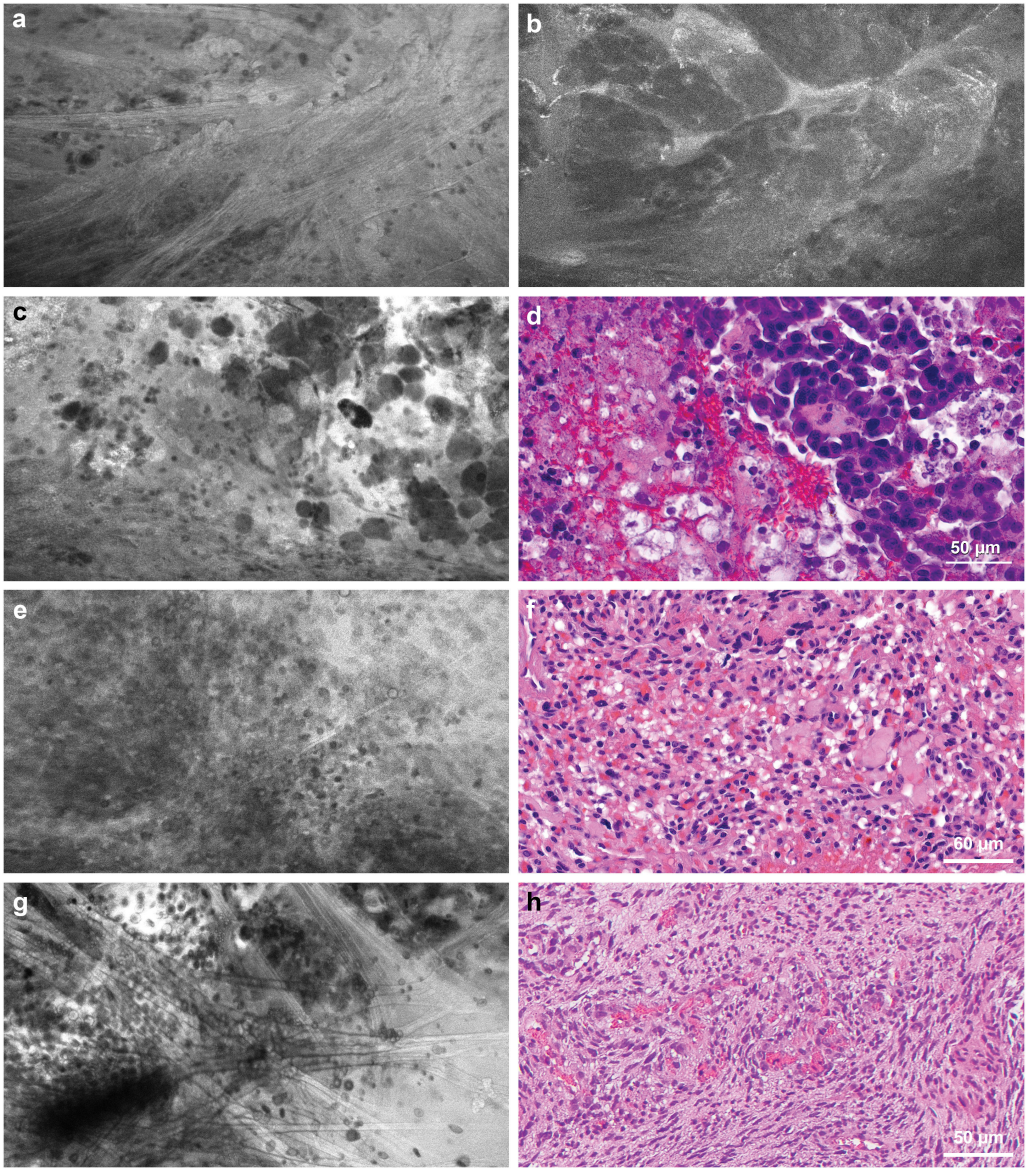
Confocal laser endomicroscopy (CLE) tumor imaging. **(a)** CLE image acquired at the dural margin of a meningioma showing sporadic tumor cells interspersed among refractile fibers that are commonly seen in dural tissue. **(b)** CLE images acquired from the underlying cortical surface showing suspicious tumor invasion by an atypical meningioma. **(c)** CLE imaging and **(d)** hematoxylin and eosin (H&E)-stained specimen showing loosely cohesive aggregate of pleomorphic cells with occasional vacuolated cytoplasm seen in metastatic breast cancer. **(e)** CLE imaging and **(f)** H&E-stained specimen showing densely packed atypical cells with vacuoles in a metastatic renal cell carcinoma of a clear cell type. **(g)** CLE image of a sellar hemangioblastoma showing remarkable microvasculature with erythrocytes in the vessel lumen, intracytoplasmic vacuoles, and atypical cells consistent with **(h)** histology of hemangioblastoma. Used with permission from Barrow Neurological Institute, Phoenix, Arizona.

Meningiomas appear moderately hypercellular, with relatively uniform cell morphology. At the dural margins, foci of tumor cells can be seen against a backdrop of refractile fibers from dural tissue (**Figure 6A**). In a case of atypical meningioma, suspicious tumor infiltration was noted in CLE images acquired from the underlying cortical surface (**Figure 6B**). CLE images of metastatic tumors showed grossly heterogeneous cellular morphology (**Figures 6C,D**). In addition, in clear cell renal carcinoma, cytoplasmic vacuoles (**Figures 6E, F**) can be recognized, although pinpointing a tumor’s etiology is not always feasible. CLE may display features unique to the tumor type in other less common intracranial tumors. CLE imaging of a sellar tumor showed the histology of classic microvasculature, intracytoplasmic vacuoles, and atypical cells consistent with hemangioblastoma (**Figures 6G, H**), precluding the need for an intraoperative frozen section biopsy that would have risked significant bleeding (26).

### 4.4 Potential impact of CLE imaging on intraoperative workflow

**Figure 7:**
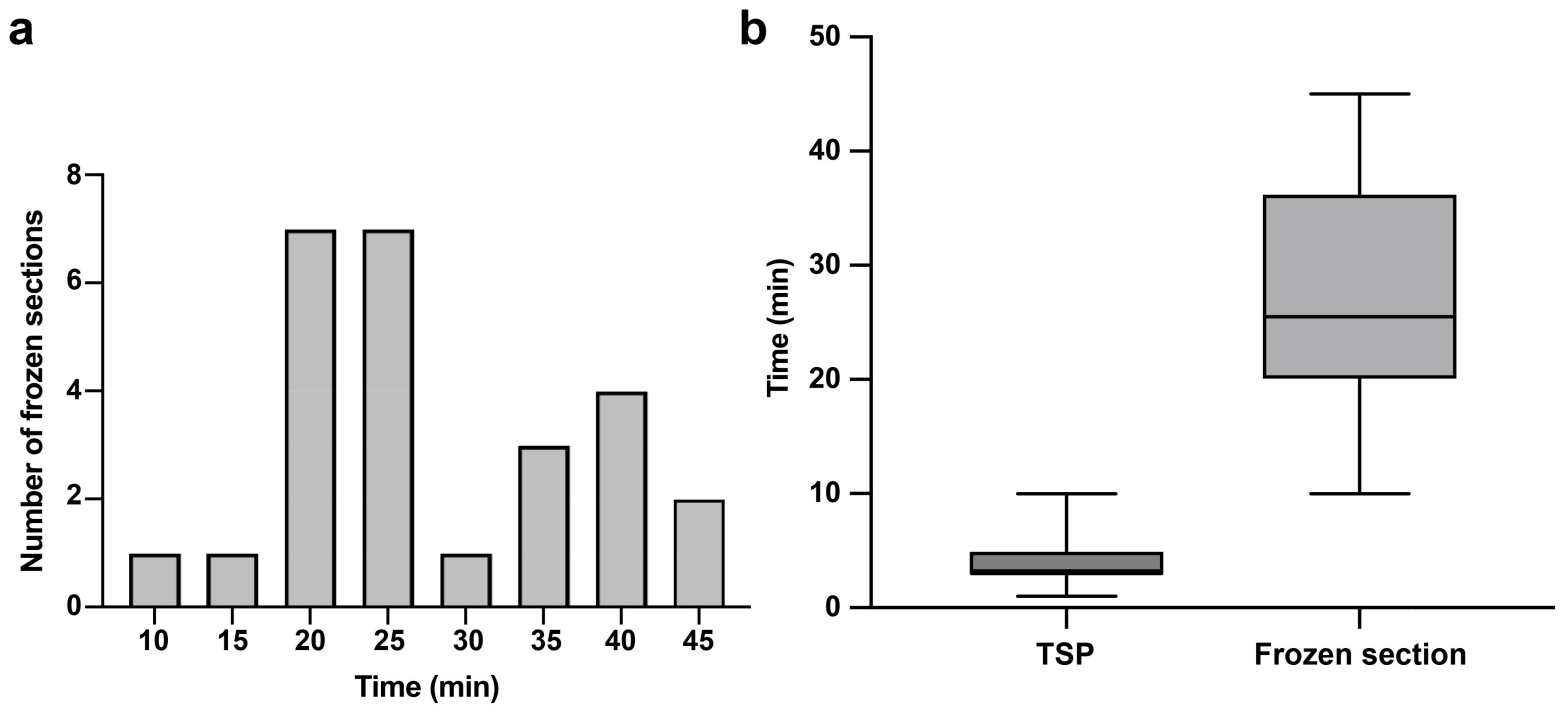
Telepathology software platform (TSP) consultation enhanced real-time communication between the neurosurgeon and neuropathologist. **(a)** Distribution of frozen section biopsy turnaround time with a mean of 28.1 min per specimen. **(b)** Bar and whisker plot showing CLE imaging time per specimen with TSP and frozen section biopsy. The horizontal line in the middle of the box indicates the median, the top and bottom borders of the box indicate the 75th and 25th percentile, respectively, and the top and bottom whisker indicate the range. Used with permission from Barrow Neurological Institute, Phoenix, Arizona.

To assess the efficiency and cost-effectiveness of intraoperative CLE imaging compared to other standard intraoperative diagnostic methods, we calculated the time and costs associated with using the CLE system during surgery. In our study, the mean (SD) CLE imaging time for each ROI with TSP consultation was 3.9 (2.2) min. The duration from tissue acquisition to receiving the frozen section report was 28.1 (9.8) min (**Figure 7A**). Our institution features a neuropathology department that processes intraoperative frozen section specimens, and the turnaround time for analyzing frozen sections could be longer in an institution lacking an on-site neuropathology department (27). The advantage in time saved with CLE imaging compared to frozen section biopsy is clear (**Figure 7B**). However, the diagnostic accuracy of CLE imaging was lower than that of frozen section biopsy at the tumor margins.

Currently, no official procedural reimbursement scheme exists for CLE. However, reimbursement for CLE could likely encourage its use and promote clinical studies to improve its efficacy. After spreading the purchase cost and TSP consultation monthly subscription over a 5-year period and assuming its use in five cases per week, the cost of CLE is approximately $1,276 per case. The cost of frozen section biopsy for three specimens per case, estimated on the basis of Medicare reimbursement, is approximately $1,050, whereas at least one vial of 5-ALA is required for wide-field fluorescence imaging and costs approximately $3,350. The cost of CLE imaging is comparable to that of frozen-section biopsies, but the reliability of CLE imaging, particularly at glioma margins, is lower than that of frozen-section biopsies. The costs presented are derived from data from a single US institution and may not be representative of those at other hospitals, either within or outside the United States.

On the other hand, CLE imaging costs less than half of a single dose of 5-ALA and provides greater insight into cellular architecture than wide-field 5-ALA fluorescence imaging. In 16 of the 50 patients in this study, wide-field 5-ALA fluorescence imaging was used alongside CLE imaging. In a recent study conducted by our group comparing simultaneous CLE and wide-field 5-ALA fluorescence imaging, we found that 5-ALA fluorescence underestimates tumor infiltration at glioma margins compared to what can be detected using CLE imaging (13). This issue arises due to various inconsistencies in 5-ALA fluorescence imaging, from administration to bleaching caused by intense operating microscope lighting, but it is especially relevant for deeply located tissue margins, where the longer working distance and lack of deeply penetrating light sources restrict illumination and thus hinder the effective activation and detection of 5-ALA-mediated fluorescence (28, 29). However, 5-ALA fluorescence has higher specificity due to its tumor-specific biology (30).

Neurosurgery is rapidly advancing, with the development of intraoperative imaging technology targeting the cellular level, such as fluorescence techniques (31) and stimulated Raman histology (32), which provides surgeons with feedback within seconds to minutes. CLE has the advantage of being a handheld microscope that produces real-time, on-demand, *in vivo* intraoperative images and can be a crucial part of the progression toward “cell surgery,” to be paired with surgical instruments at a similar level. In many situations, it could supplant the need for a frozen section, thereby significantly speeding up surgery and allowing for rapid, broad interrogation of the lesion and surgical bed, especially where tumor remnants or invasion are suspected. CLE may have the potential to be paired with rapid tumor tissue molecular analysis, enabling preferential selection of tissue harvest sites. Future integration of artificial intelligence image discrimination algorithms could improve functionality and incorporate built-in image stabilization. CLE would then play a significant role in the resection of primary invasive brain tumors.

CLE augmented by telepathology may significantly affect the operative workflow by reducing the time needed for an intraoperative histological diagnosis. The intraoperative interaction through TSP between the neurosurgeon and neuropathologist represents a substantial improvement in communication. Previous studies show that reliable interpretation of images can be accomplished by pairing a CLE-experienced neurosurgeon and neuropathologist (13). CLE provides consistent histopathological information when imaging the tumor core. However, CLE imaging of the glioma tumor margin remains challenging due to its lack of specificity. Recent research suggests that CLE’s sensitivity for detecting tumor cells at the glioma margin may exceed that of 5-ALA (13).

## 5 Conclusions

Indistinct infiltrating glioma margins present well-known challenges for discrimination, interpretation, and surgical resection. The goal of removing every invading cell is impractical given the highly infiltrative nature of the disease. However, if the goal is to optimize the resection of nests of cells that remain at the tumor resection site or in questionable locations, then rapidly scanning specific areas with CLE would provide a distinct advantage and enable “optical biopsies.” Large prospective clinical trials that assess the extent of resection and survival benefit will help delineate whether the current FNa-based CLE technology becomes an essential neuro-oncological surgical tool. With increasing intraoperative experience and improved specificity of the current fluorescence system ranges (e.g., with the development of new fluorophores or improved use and detection of FNa), CLE may represent a significant time- and cost-effective addition to our current tools for intraoperative guidance.

## Authors’ note

This study was presented in part as a poster at the 2024 Congress of Neurological Surgeons Annual Scientific Meeting; Houston, Texas; 28 September 2024−2 October 2024.

## Data Availability

The raw data supporting the conclusions of this article will be made available by the authors, without undue reservation.
